# Integrated proteomics and metabolomics analysis of lumbar in a rat model of osteoporosis treated with Gushukang capsules

**DOI:** 10.1186/s12906-022-03807-7

**Published:** 2022-12-15

**Authors:** Ruohui Lin, Bingying Xie, Lihua Xie, Jirong Ge, Shengqiang Li

**Affiliations:** 1 Basic Research Institute, Fujian Academy of Chinese Medical Sciences, Fuzhou, 350003 Fujian China; 2 Fujian Key Laboratory of Integrated Traditional Chinese and Western Medicine for the Prevention and Treatment of Osteoporosis, Fuzhou, 350003 Fujian China

**Keywords:** Proteomics, Metabonomics, Gushukang capsules, Osteoporosis, Integration, Bioinformatics

## Abstract

**Background:**

Gushukang (GSK) capsules are a Chinese patented medicine that is widely used in clinics for the treatment of osteoporosis (OP). Animal experiments have revealed that the bone mineral density of osteoporotic rats increase after treatment with GSK capsules. However, the specific mechanism and target of GSK in the treatment of osteoporosis are unclear. Further studies are needed.

**Methods:**

Metabolomics (GC/MS) and proteomics (TMT-LC-MC/MC) with bioinformatics (KEGG pathway enrichment), correlation analysis (Pearson correlation matrix), and joint pathway analysis (MetaboAnalyst) were employed to determine the underlying mechanisms of GSK. The differential expression proteins were verified by WB experiment.

**Results:**

The regulation of proteins, i.e., Cant1, Gstz1, Aldh3b1, Bid, and Slc1a3, in the common metabolic pathway of differential proteins and metabolites between GSK/OP and OP/SHAM was corrected in the GSK group. The regulation of 12 metabolites (tyramine, thymidine, deoxycytidine, cytosine, L-Aspartate, etc.) were differential in the common enrichment metabolic pathway between GSK /OP and OP/SHAM. Differential proteins and metabolites jointly regulate 11 metabolic pathways, such as purine metabolism, pyrimidine metabolism, histidine metabolism, beta-alanine metabolism, and so on.

**Conclusion:**

GSK may protect bone metabolism in osteoporotic rats by affecting nucleotide metabolism, amino acid metabolism, and the immune system.

**Supplementary Information:**

The online version contains supplementary material available at 10.1186/s12906-022-03807-7.

## Background

Osteoporosis (OP) is a systemic metabolic disease characterized by low bone mass, microstructural damage of bone tissue, decreased bone strength, and increased bone fragility [1]. Its clinical manifestations include primarily pain and spinal curvature [2]. For thousands of years, traditional Chinese medicine (TCM) has been used in both China and other Asian countries [3]. At present, TCM has obvious curative effects and benefits in the treatment of osteoporosis. These can better improve the side effects and safety of standard treatment. TCM formulas not only reduce bone loss by decreasing bone resorption but also increase bone formation in the multicomponent and multitarget pattern [4]. It can also regulate the overall function of the human body and relieve back pain and lumbago [5].

The GSK capsule is a Chinese patented medication listed in the Chinese Pharmacopoeia that is used for treating osteoporosis. Its main effective components include *Epimedii folium*, *Rehmannia radix praeparata*, *Astragalus membranaceus (Fisch.)Bge.* and *Drynaria rhizoma*. Chinese scholars have established an HPLC-CAD method to determine the content of the main components of the GSK capsules, which provides a reference for the quality control of the GSK capsules [6]. There is sufficient evidence that the main components of the GSK capsules, such as Epimedium and Drynaria, have antiosteoporosis function. Epimedium has been proved to improve bone mineral density or bone metabolism [7]. Rhizoma Drynariae can promote osteogenesis differentiation by activating the Wnt/β-catenin signaling pathways, which play a role in antiosteoporosis activity [8]. Animal experiments have shown that *Rehmannia radix praeparata* could significantly increases the expression levels of Akt1 and MAPK1 mRNA in the bone tissue to increase bone density [9]. Astragalus saponin IV (AS- IV) extracted from *Astragalus membranaceus (Fisch.)Bge.*, promotes the osteogenic differentiation of the bone marrow mesenchymal stem cells via the MIR-21/NGF/BMP2/Runx2 pathway [10]. GSK capsules provide convenient administration, stable curative effects, and few side effects. In the guideline for the clinical diagnosis and treatment of primary osteoporosis in China, GSK is selected as the recommended Chinese patent medicine [11], and it is widely used in the clinical settings [12]. Clinical research shows that GSK is effective in the treatment of primary osteoporosis, by adjusting the whole-body function. GSK can inhibit the formation of osteoclasts and stimulate the formation of osteoblasts in ovariectomized mice [13]. It also significantly increases the production of vitamin D and calcium [14]. The BMP/Smad signaling pathway is important in bone remodeling. Interestingly, a study found that GSK significantly enhanced the BMP-2/Smad signaling pathway and improved bone microstructure by upregulating relevant osteogenic factors in osteoporotic rats [15]. Furthermore, H-type blood vessels have shown the ability to induce angiogenesis and bone formation [16]. Recent studies demonstrated that GSK can enhance hypoxia-inducible factor-1α to induce H-type vascular formation as well as bone formation [17].

**Fig. 1:**
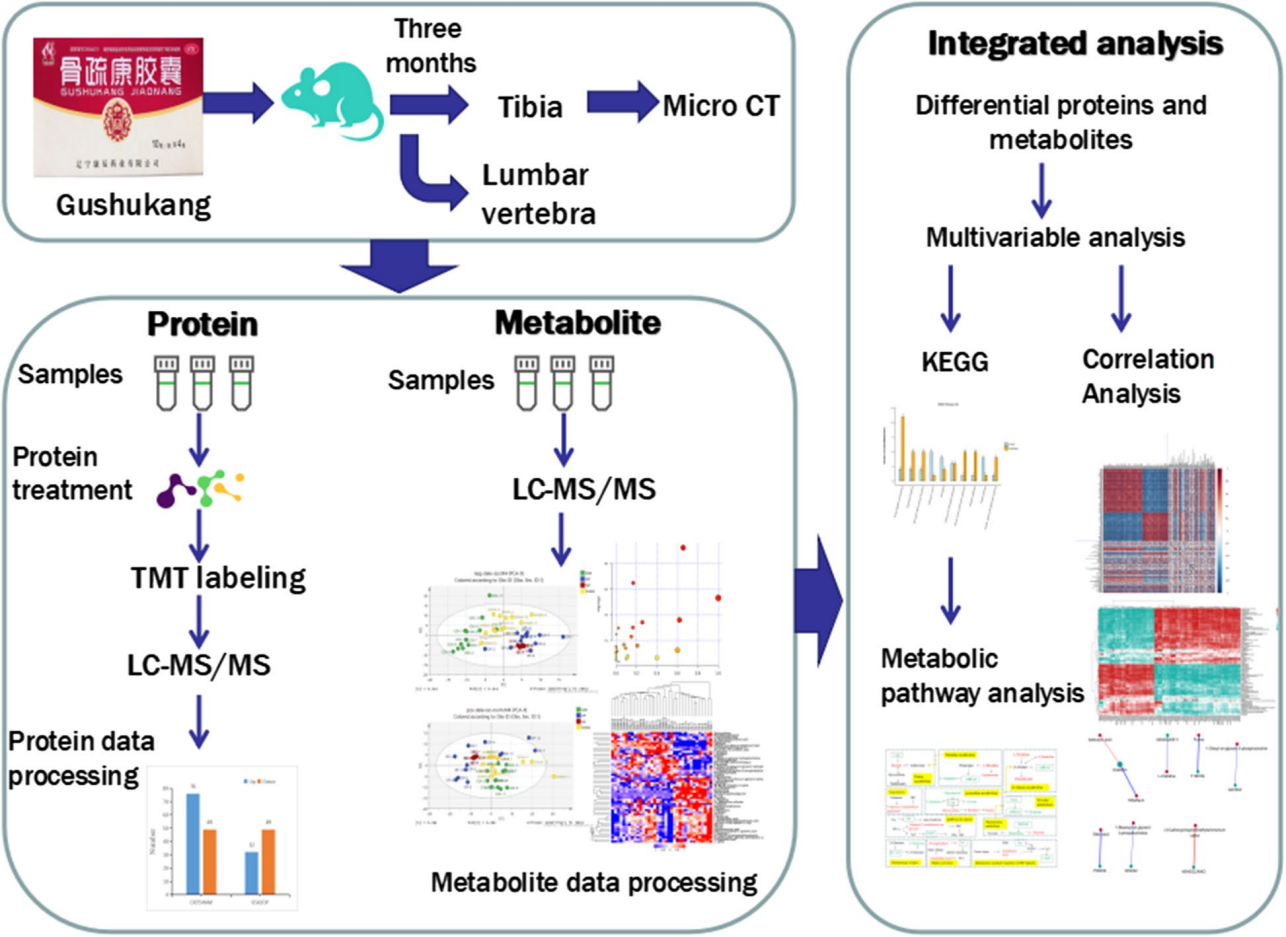
A graphic abstract of the article

In recent years, multiomics integrated analysis technology has been widely used to study various disease states [18]. Integrating multiomic data analyses can make up for data loss, noise, and other factors in single omic data analysis; integrate various interactions isolated at the gene level or protein level, various metabolic, and regulatory pathways; and jointly clarify the overall state of the biological system [19]. The combined application of proteomic and metabolomic technology has undeniable advantages [20]. Data from metabolomic analysis can provide information on molecular function for the results of proteomic research and provide clues to explore the regulatory relationship between them. The resulting protein–metabolite interaction network intuitively shows the involved, maladjusted pathways in the disease state or the overall changes after drug treatment. Proteomics and metabolomics provide us with vast information of differential proteins and metabolites. In turn, this provides a new research strategy for the target research of TCM in the treatment of diseases. However, the specific mechanism and target of GSK in OVX rats remain unclear. Therefore, based on the integrated analysis of proteomics and metabolomics, this study used GSK capsules to further explore the mechanism of GSK in the treatment of osteoporotic rats. The research flowchart is shown in Fig. 1.

## Methods

### Drugs and reagents

Gushukang capsule was supplied by Liaoning Kangchen Pharmaceutical Co., Ltd. (Z20060270) (Dandong, China). BCA quantitative kit was purchased from Beyotime Biotech Inc. (Shanghai, China). SDT lysate, TMT labeling kit were from Thermo Fisher Scientific Inc. (Waltham, USA). Acetonitrile was from Merck & Co Inc. (Kenilworth, USA), Ammonium acetate was from Sigma (Aldrich, USA). Anti-Slc1a3 rabbit polyclonal antibody [EPR12686] was from Abcam Plc ((Cambridge, USA), anti-Aldh3b1 rabbit polyclonal antibody [TA323583S] was from OriGene Technologies, Inc. (Rockwell, USA), anti-Gstz1 rabbit polyclonal antibody [No.Ag6676], anti-Cant1 rabbit polyclonal antibody [No.12164–1-AP], anti-Bid rabbit polyclonal antibody [No.10988–1-AP] were from Proteintech Group, Inc. (Rosement, USA). Anti-β-actin rabbit polyclonal antibody [bs-0061R], Goat anti-rabbit IgG H&L HRP [bs-0295G-HRP] were from Beijing Biosynthesis Biotech Co., Ltd. (Beijing, China). MonPro™ ECL ultrasensitive substrate pro [PW30701] was from Monad Biotech Co., Ltd. (Wuhan, China).

### Animal model establishment

All experimental procedures complied with the revised Animals (Scientific Procedures) Act 1986 and the ARRIVE (Animal Research: Reporting of In Vivo Experiments) guidelines, and were approved by the Animal Ethics Committee of Fujian Academy of Traditional Chinese Medicine (Fujian, China) (FJATCM-IAEC2018034). Anesthesia methods that we employed in the experiment complied with American Veterinary Medical Association (AVMA) Guidelines for the Euthanasia of Animals (2020). Thirty-six, 3-month-old female SPF Sprague Dawley rats were obtained from Shanghai Slake Laboratory Animal Co., Ltd. (Shanghai, China) [SCXK(Shanghai)2007–0005], and raised to 6 months old in the Experimental Center of Comparative Medicine, Fujian Academy of Chinese Medical Sciences (Fujian, China) [SYXK(Fujian)2009–000]. All rats were maintained in a 12-h light/dark cycle, with controlled temperature (22 °C–24 °C) and humidity (50–60%). After 1 week of adaptive feeding, 6-month-old rats (*n* = 36) were randomly divided into three groups: the GSK group (*n* = 12), the model group (n = 12), and the sham operation group (n = 12). An osteoporosis model was established by removing the bilateral ovaries of rats. Approximately 1 g of adipose tissue adjacent to the ovary was removed in the sham group [21]. Intramuscular injection of penicillin at 800,000 units/day was used to prevent infection for 3 days after the ovariectomy.

### GSK treatment

The GSK capsules comprise seven medicinal compositions as shown in Table 1. GSK capsules were opened and mixed with normal saline, and administered by gavage once a day (0.32 g/(kg × d)). Both the sham group and model group were given an equal volume of normal saline once a day for 12 weeks. After 12 weeks of treatment, rats in each group were anesthetized with 2% pentobarbital sodium. The first and second lumbar vertebrae were separated and quickly put into liquid nitrogen. The left tibia was used for micro-CT.

**Table 1:** Ingredients of Gushukang capsules

Components	Part used	Rate(%)
*Epimedium brevicornu Maxim*.	Leaf	17.52
*Rehmannia glutinosa Libosch*.	Root tuber	23.19
*Drynaria fortunei (Kunze) J.Sm*.	Rhizome	11.59
*Astragalus membranaceus (Fisch.)Bge*.	Root	17.52
*Salvia miltiorrhiza* * Bge*.	Root/Rhizome	11.59
*Auricularia auricula (L.ex Hook.) Underw*	Fruiting body	9.29
	Seed	9.29

### Micro-CT bone analysis

Micro-computed tomography (micro-CT) scanning and morphometric analysis were performed on a micro-CT imaging system (ZKKS-MCT-Sharp, Zhongkekaisheng Co., China), which operated at a voltage of 70 kV and an electric current of 100 μA. The voxel size after reconstruction was 10 × 10 × 10 μm. Based on the micro-CT results, three-dimensional images were reconstructed by micro-CT reconstruction. The bone morphometric parameters of the left tibia were measured by analysis software, including bone mineral density (BMD), bone volume/total volume (BV/TV), trabecular separation (Tb.Sp) and trabecular number (Tb.N). The data are displayed as means ± standard errors of the mean (SEM). All statistical analyses were performed on Prism 8.0 (GraphPad Prism Software, USA).

### Proteomic sample processing

Lumbar samples were added with appropriate SDT lysate. After boiling in a water bath, the supernatant was centrifuged to extract the protein. The lumbar vertebrae proteins of every four rats were combined into one sample for proteomic analysis. Protein was quantified using the BCA method. An appropriate amount of protein from each sample was trypsinized using the filter-aided proteome preparation (FASP) method [22], and the peptides were quantified (OD280). Approximately 100 UG peptide segments were taken from each sample and labeled according to the instructions of the TMT labeling kit.

### Liquid chromatography tandem mass spectrometry (LC-MS/MS)

Each fractionated sample was separated by the HPLC liquid-phase system EASY-nLC (Thermo Fisher Scientific, USA) with nanoliter flow rate. After chromatographic separation, the samples were analyzed by a QExactive mass spectrometer (Thermo Fisher Scientific, USA). Positive ion detection method, parent ion scanned range 300–1800 m/Z. Mass charge ratio collection method of polypeptide and polypeptide fragments: collect 20 fragment maps (MS2 scan, HCD) after each full scan.

### Proteomic data analysis

The original data was analyzed by LC-MS/MS software, Mascot 2.2, and Proteome Discoverer 1.4. The protein database is UniProt_ Rattus_ norvegicus_36107_ 20,190,524. The quantification method was based on the median quantitative value of the unique peptide. The maximum number of missed cuts is two. The variable modifications were oxidation (m) and TMT6/10 plex (y). The fixed modifications include carbamide methyl (c), TMT6/10 plex (N-term), and TMT6/10 plex (k). The screening criteria of credible peptides were ≤ 0.01.

### Screening of differential proteins

Differentially expressed proteins were screened using a multiplier variation that was higher than 1.2 times (up to 1.2 times or less than 0.83) and with a *p*-value of < 0.05. Simultaneously, hierarchical clustering was used to analyze differentially expressed proteins in the comparison group, after which obtained data were displayed on a heatmap.

### Metabonomic sample processing

Approximately 80-mg samples were added to 200-ul water. Samples were then homogenated with an MP homogenizer and vortexed for 60 s. Approximately 800-ul methanol acetonitrile solution (1:1, v/v) was added, and the mixture was vortexed for another 60 s. The suspension was ultrasonicated at low temperature for 30 min twice and placed at − 20 °C for 1 h to precipitate the protein. The suspension was then centrifuged at 4 °C, 14000 RCF, for 20 min. Finally, the supernatant was freeze-dried and stored at − 80 °C.

### Gas chromatography mass spectrometry

The samples were separated in an HILIC column by 1290 Infinity LC (Agilent, USA) ultra-performance liquid chromatography (UHPLC). The column temperature was 25 °C, and the flow rate was 0.3 ml/min. The mobile phase consisted of either water + 25 mM ammonium acetate + 25 mm ammonia water (A) or acetonitrile, gradient elution (B). QC samples were inserted into the sample queue to evaluate both the stability of the system and the reliability of experimental data. The positive and negative ion modes of electrospray ionization (ESI) were detected. Samples were separated by UHPLC and then analyzed by a TripleTOF 5600 mass spectrometer (AB SCIEX). Second-order mass spectrometry was obtained by information-dependent acquisition (IDA) with the high sensitivity mode. The following settings were used: declustering potential of ±60 V (positive and negative modes) and collision energy of 35 ± 15 eV. IDA was set as follows: Exclude isotopes within 4 Da Candidate ions to monitor per cycle: 6.

### Metabolomic data processing

The original data was converted into an mzXML format by ProteoWizard. Peak alignment, retention time correction, and peak area extraction were performed by the XCMS program. Metabolite structure identification uses accurate mass matching (< 25 ppm) and secondary spectrum matching methods while searching the laboratory’s self-built database. After pareto-scaling pretreatment, the data was analyzed using unsupervised principal component analysis (PCA).

### Screening of differential metabolites

We used the PLS-DA data model to further screen differential metabolites among our three groups to obtain variable importance in projection (VIP) under both positive and negative ion mode conditions. Afterward, metabolites with a multidimensional statistical analysis of VIP > 1 and univariate statistical analysis *p*-value < 0.05 were selected to identify potential metabolites with significant differences. Subsequently, MetaboAnalyst 5.0 was used to analyze metabolic pathways involved in differential metabolites, and then to evaluate their importance. When the impact value of the understudied metabolic pathways was > 0.2, we considered these pathways as potential target metabolic pathways in this experiment.

### Integration analysis of differential proteins and metabolites and the common pathway enrichment

For pathway enrichment analysis, dysregulated metabolites and proteins were selected and mapped to the Kyoto Encyclopedia of Genes and Genomes (KEGG) database [23–25] (http://home.jp/kegg/).

### Correlation analysis between metabolomics and proteomics

The correlation coefficient between significantly differential proteins and metabolites was calculated using Pearson’s correlation, after which results were displayed in the form of a correlation coefficient matrix heatmap (the software was R3.4.2). To intuitively reflect the difference in expression patterns of the significant differences obtained between differential protein and metabolites, we then conducted Pearson’s correlation hierarchical clustering (the software was a R3.4.2 heatmap). Subsequently, all nodes were loaded onto Cytoscape for network construction based on the correlation data. Afterward, we screened metabolites and proteins with significant differences at critical nodes in the network.

### Detection of differential proteins by western blotting

Total protein in the lumbar was extracted with RIPA buffer. A BCA protein assay kit was used for concentration measurement. 4–12% PAGE gel was used for electrophoresis. The protein was transferred to PVDF membrane using eBlot L1 Fast Wet Transfer System (Genescript biotech Corp., China). After blocking with 5% skim milk dissolved in 1× Tris-buffered saline and 0.1% Tween 20, the PVDF membranes were cut prior to hybridisation with antibodies. The cropped PVDF membranes were incubated with the diluted primary antibody [Anti-Slc1a3 (1∶1000 dilution), anti-Aldh3b1 (1∶1000), anti-Cant1 (1∶1000), anti-Gstz1(1∶1000), anti-Bid (1: 1000) and anti-β-actin (1∶4000) antibodies] at 4 °C overnight. The membrane was then washed with 1 × TBST three times and incubated with secondary antibody for 1 h. After washing three times with 1 × TBST, MonPro™ ECL ultrasensitive substrate pro was added dropwise and exposed in the dark room for 1 min. Results were visualized using the Fluor Chem M Digital Darkroom (ProteinSimple Ltd., USA).. Stained bands were analyzed with Image J to calculate the gray value. Gray values of aim protein/β-actin were taken as the relative value of differential protein expression in the lumbar. All statistical analyses were performed on Prism 8.0.

## Results

### Effect of the GSK capsules on bone trabecular in osteoporotic rats

**Fig. 2:**
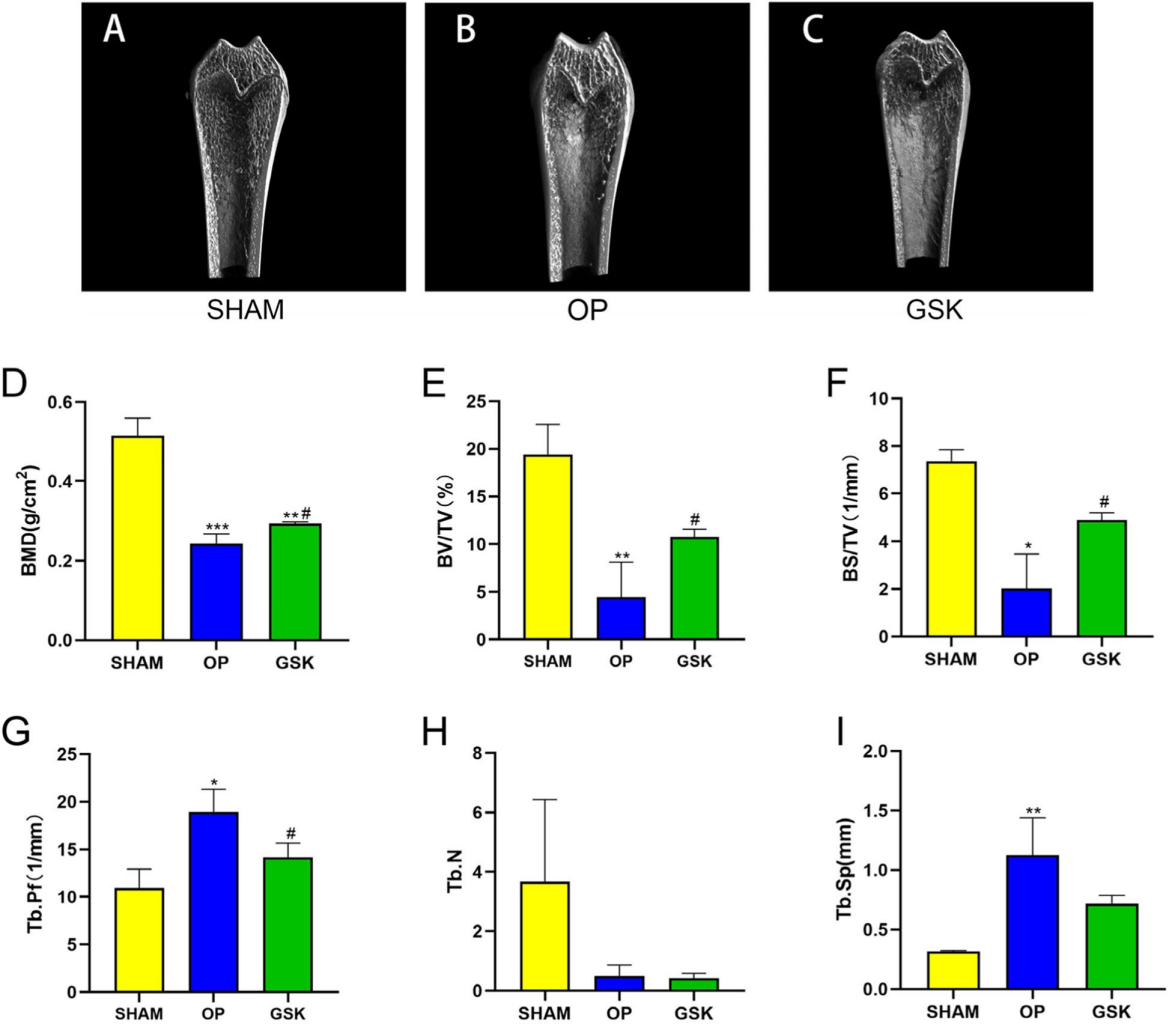
Bone plain scan and bone mineral density (BMD) of the trabecular bone in the proximal left tibia. Representative plain scan images of the proximal tibia were shown (sagittal) in **A** SHAM, **B** OP, and **C** GSK. The BMD indices and parameters of micro CT are shown as the mean ± SEM (*n* = 3 per group). ***p < 0.001 vs. the sham group; **p < 0.01 vs. the sham group; *p < 0.05 vs. the sham group; ^#^ *p* < 0.05 vs. the OP group

The proximal end of the left tibia was evaluated with a micro-CT plain scan (Fig. 2A, B, C). From the analysis results of bone trabecular morphological parameters, the BMD (*P* < 0.001, Fig. 2D), BV/TV (*P* < 0.01, Fig. 2E), BS/TV (*P* < 0.05, Fig. 2F) of the OP group decreased significantly, while Tb.Pf (*P* < 0.05, Fig. 2G) and Tb.Sp (*P* < 0.01, Fig. 2I) increased significantly compared with the SHAM group. In the OP group, Tb.N tended to decrease, but there was no statistical difference (*P* > 0.05, Fig. 2H).

In the GSK group, the BMD, BV/TV and BS/TV increased significantly compared with the OP group (*P* < 0.05, resptively, Fig. 2D, E, F), while Tb.Pf decreased significantly (*P* < 0.05, Fig. 2G). Tb.Sp showed a decreasing trend in GSK group, but there was no significant difference compared with OP group (*P* = 0.09, Fig. 2I).

### Results of differential protein analysis

**Fig. 3:**
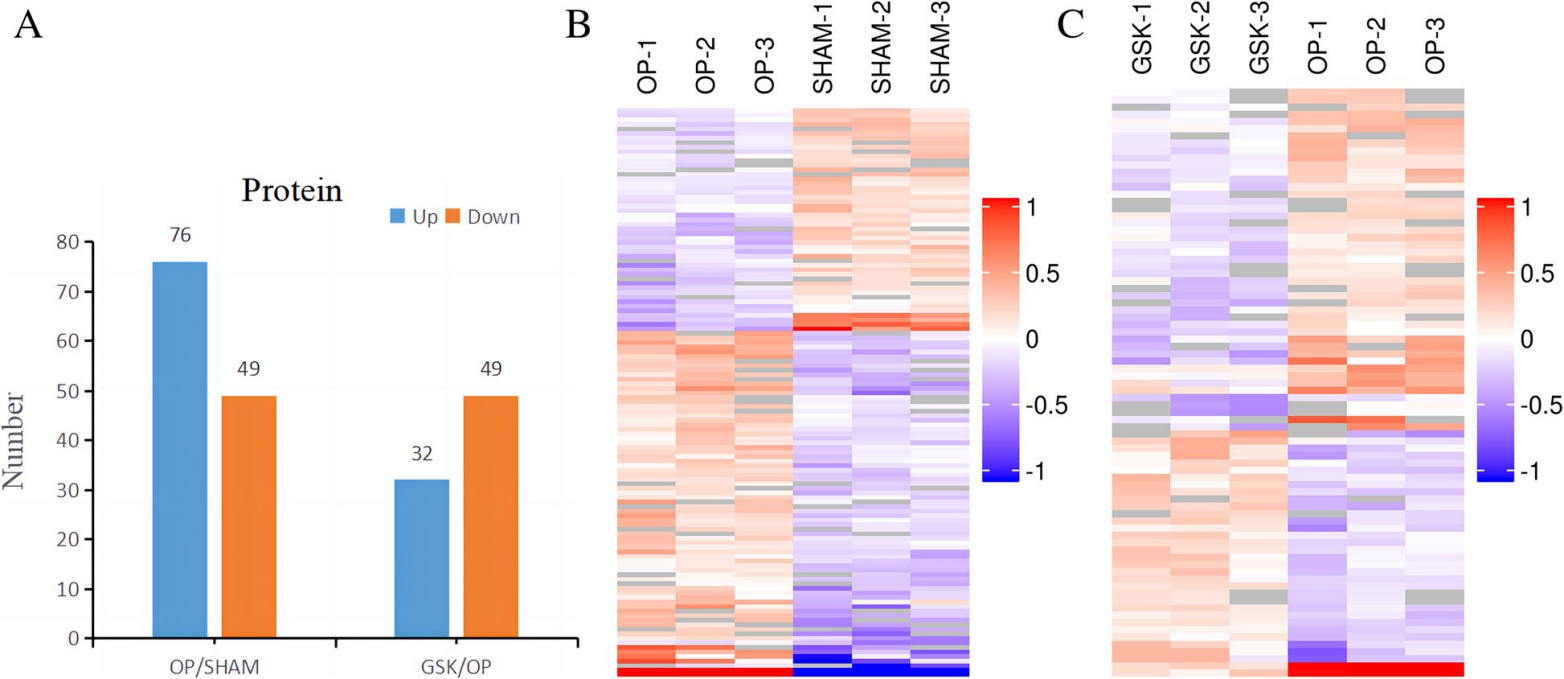
Different expression trends of differential proteins in each group (**A**). Cluster analysis of differentially expressed proteins in each group (**B**) (**C**). Hierarchical clustering results were represented as a tree heat map. Each row represents a protein, each column represents a set of samples, and Log2expression values of significant differentially expressed proteins in different samples are presented in different colors on the heat map. Furthermore, red represents significantly upregulated proteins, blue represents significantly downregulated proteins, and gray represents no quantitative protein information

We identified 3614 proteins in the three groups. Subsequently, we then compared the differentially expressed proteins between these groups using the differential protein screening criteria mentioned in the above experimental method (Fig. 3), followed by a statistical analysis of the expression of differential proteins among GSK, OP, and SHAM groups. As observed, differentially expressed proteins in OVX rats were corrected in the GSK group (top 5) (Table 2). Table 3 lists the five proteins with the most significant upregulation and the five with the greatest downregulation in the OP group.

**Table 2:** Statistical results of upregulated and downregulated proteins between GSK and OVX (top 5)

Uniprot ID	Protein name	Gene name	GSK VS OVX		
			***P*** value	Difference multiple	Regulation
A0JPP1	Dr1-associated corepressor	Drap1	0.026	1.749	Up
G3V7U0	Cysteine and glycine-rich protein 3	Csrp3	0.048	1.527	Up
D3ZES7	Plexin A4	Plxna4	0.044	1.404	Up
M0RDF2	Ig-like domain-containing protein	–	0.031	1.392	Up
D4A7U8	Myozenin 1	Myoz1	0.041	1.383	Up
Q5BJX0	N-terminal Xaa-Pro-Lys N-methyltransferase 1	Ntmt1	0.003	0.833	Down
P0C0R5	Phosphoinositide 3-kinase regulatory subunit 4	Pik3r4	0.026	0.833	Down
Q5U206	Calmodulin-like protein 3	Calml3	0.008	0.833	Down
P70582	Nuclear pore complex protein Nup54	Nup54	0.031	0.832	Down
A0A0G2K6H2	Maleylacetoacetate isomerase	Gstz1	0.021	0.832	Down

**Table 3:** Statistics of upregulated and downregulated proteins between OVX and SHAM (top5)

Uniprot ID	Protein name	Gene name	OVX VS SHAM		
			***P*** value	Difference multiple	Regulation
D3ZAT0	RCG32168	Svs3b	≤0.001	6.329	Up
O88753	Epsilon 2 globin	Hbe2	≤0.001	5.373	Up
B2RYM6	Zc3hc1 protein	Zc3hc1	0.010	2.493	Up
Q6AY07	Fructose-bisphosphate aldolase	Aldoart2	0.039	2.284	Up
E9PTV9	Glyceraldehyde-3-phosphate dehydrogenase	–	0.002	2.071	Up
P62762	Visinin-like protein 1	Vsnl1	0.012	0.831	Down
P43278	Histone H1.0	H1f0	0.003	0.830	Down
F1LST1	Fibronectin	Fn1	0.043	0.828	Down
D3ZPV2	RCG43880	Tktl1	0.033	0.821	Down
P14480	Fibrinogen beta chain	Fgb	0.004	0.819	Down

### Results of differential metabolite analysis

**Fig. 4:**
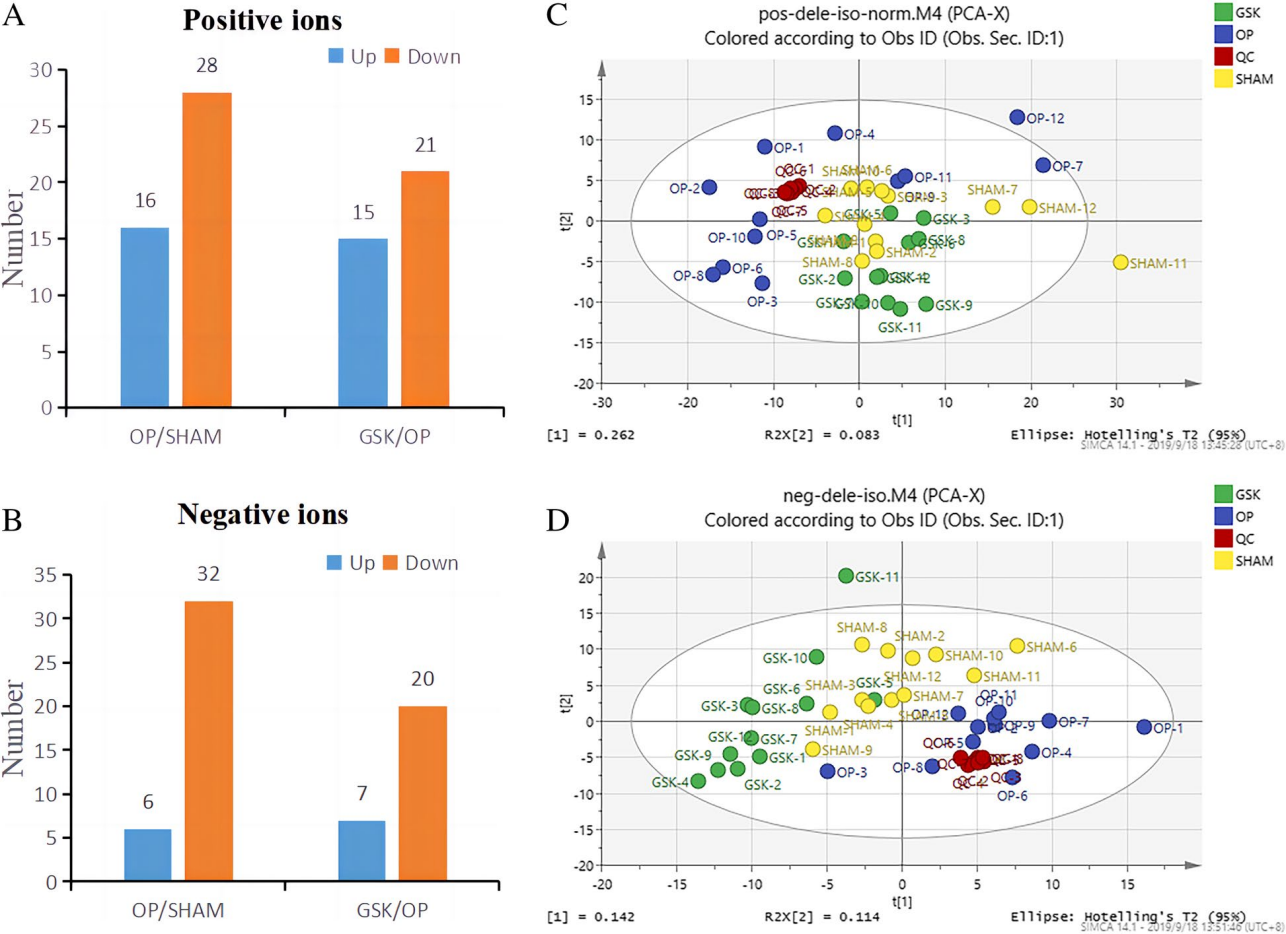
Differing expression trends of differential metabolites in each group (**A**) (**B**). PCA map showing metabolomics results in positive and negative ion modes (**C**) (**D**). Each point in the figure represents a sample. Different colors represent different groups. Furthermore, closer samples represent closer metabolite expression patterns, which means more minor differences between the groups. We used the PCA method to evaluate the aggregation degree and the overall distribution trend of samples within the group

**Fig. 5:**
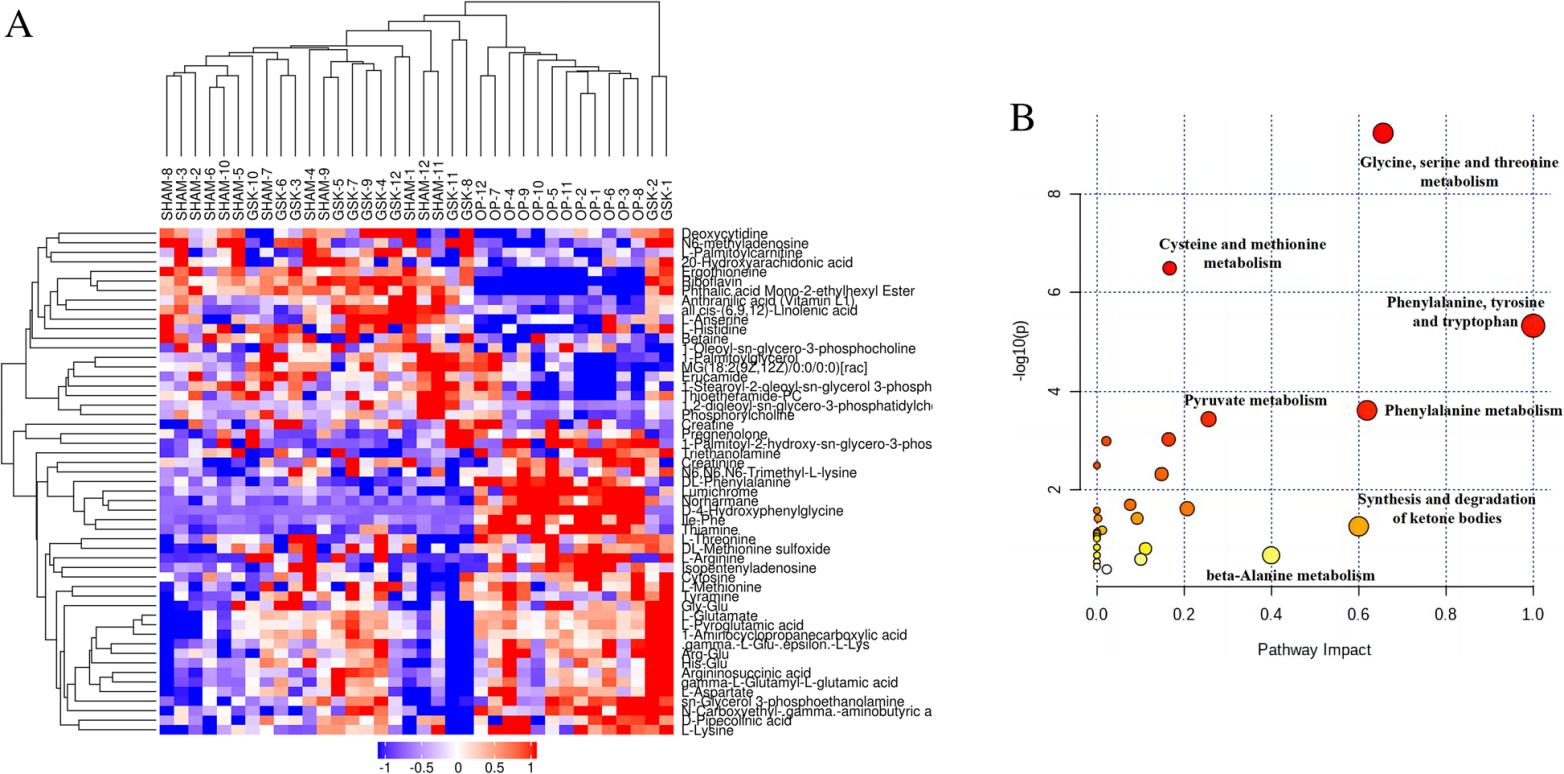
Cluster analysis showing differentially expressed metabolites in the three groups (**A**). Map showing significant metabolic pathways involved in differential metabolites (**B**)

Figure 4A and B shows the number of abnormal metabolites in positive and negative ion modes. PCA results indicated that the QC and other samples were well aggregated within the group, indicating that the overall quality of the experimental data met the analysis requirements. Significant differences were observed in metabolite levels among the GSK, SHAM, and OP groups (Fig. 4C, D). The significantly different metabolites (Fig. 5A) were mainly involved in the following pathways (Fig. 5B): phenylalanine, tyrosine, tryptophan, glycine, serine and threonine metabolism, phenylalanine metabolism, synthesis and degradation of ketone bodies, beta-alanine metabolism, cysteine and methionine metabolism, and pyruvate metabolism.

### The number of common metabolic pathways of differential proteins and differential metabolites

**Fig. 6:**
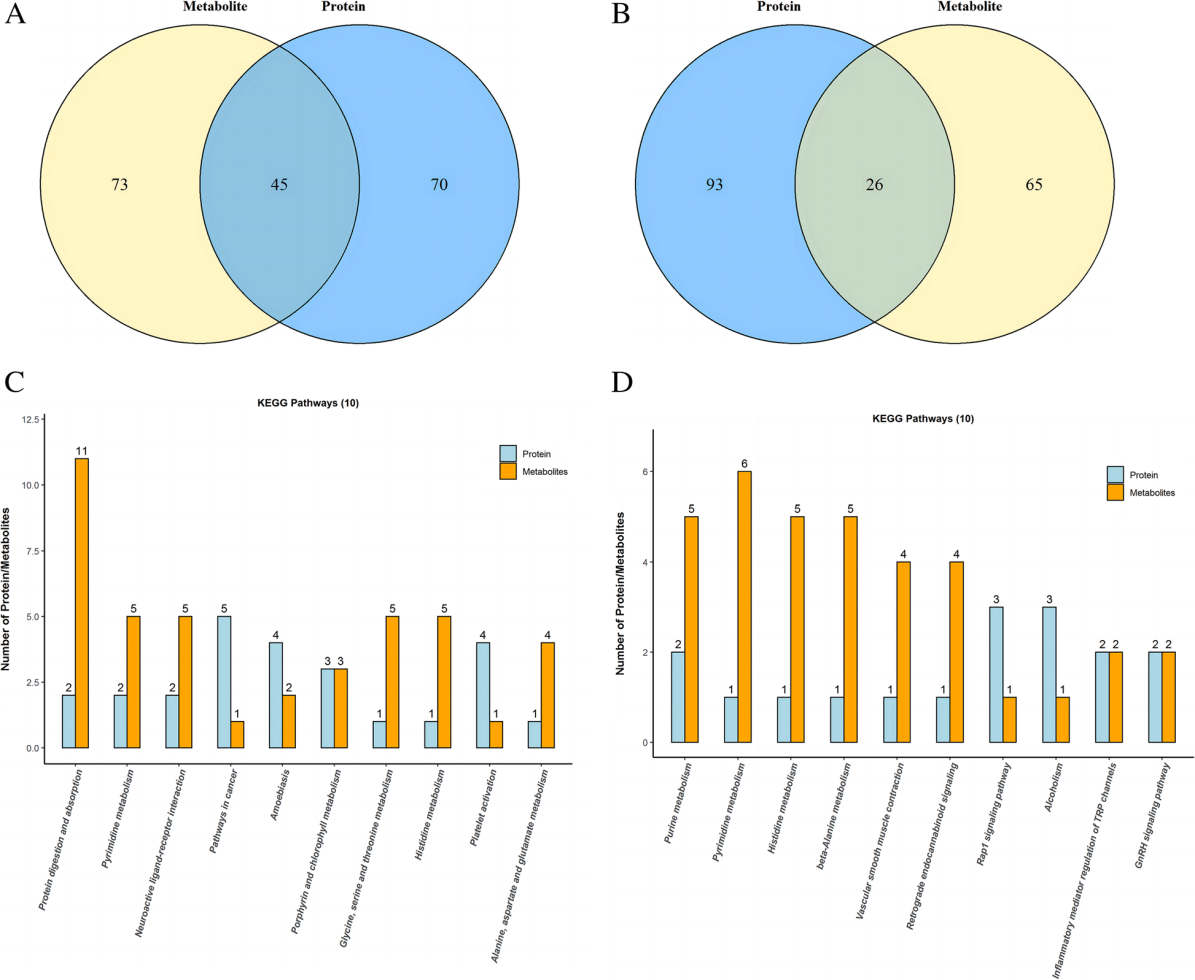
Venn diagrams showing the number of metabolic pathways involved in differential proteins and differential metabolites in GSK/OP and OP/SHAM (**A**) (**B**). The number of differential proteins and differential metabolites contained in these metabolic pathways (TOP 10) (**C**) (**D**)

Differential proteins between GSK and OP groups were involved in 70 metabolic pathways (Fig. 6A). However, although these differential metabolites were involved in 73 metabolic pathways, two parts jointly participated in 45 pathways. The results also showed that differential proteins between OP and SHAM groups were involved in 93 metabolic pathways (Fig. 6B). Nevertheless, while differential metabolites were involved in 65 metabolic pathways, two parts were jointly involved in 26 pathways. Figure 6C, D shows the top 10 pathways.

### Correlation analysis of significantly different proteins and metabolites

**Fig. 7:**
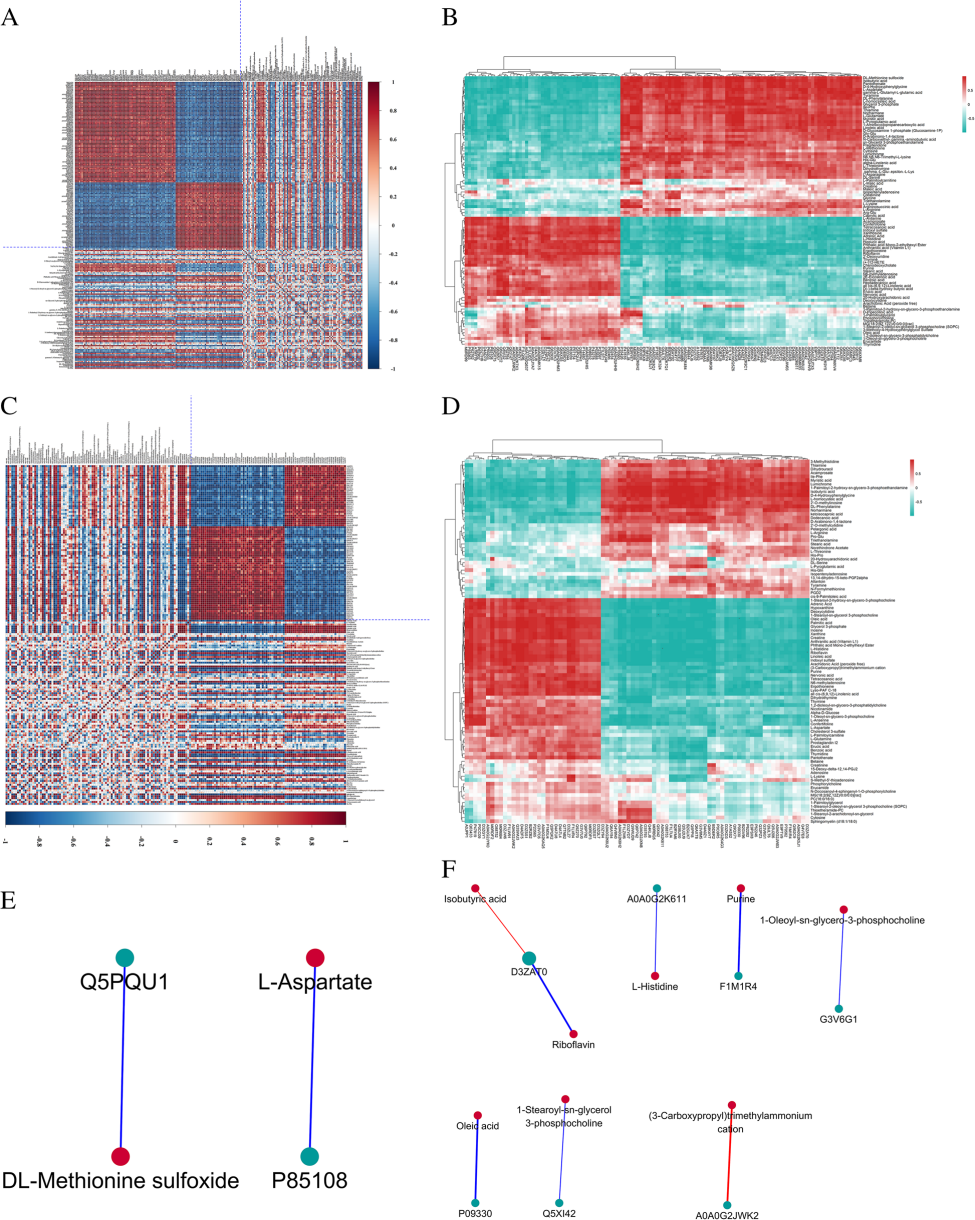
Matrix heatmap showing the correlation coefficient matrix of significantly different proteins expressing significantly different metabolites (**A**) (**C**). *r* was identified between −1 and + 1. The correlation coefficient (*r*) for proteins and metabolites is indicated using colors. While *r* > 0 indicates a positive correlation and is represented in red, *r* < 0 indicates a negative correlation and is represented in blue. Darker colors indicate stronger correlations. Hierarchical clustering heatmap showing Pearson’s correlation analysis of differential proteins and metabolites (**B**) (**D**). Each row indicates a significantly different metabolite, whereas each column indicates a significantly different protein. The correlation analysis network between GSK/OP and OP/SHAM for significantly different proteins and metabolites (**E**) (**F**)

We constructed a correlation network of proteins and metabolites (|*r*| ≥ 0.5 and *P* < 0.05) (Table 4), used matrix heatmap and hierarchical heatmaps to represent their correlation coefficients (Fig. 7A-D), and found that the metabolites involved in histidine metabolism and β-alanine metabolism were strongly related to the differential proteins. In the correlation network of GSK/OP, seven proteins and eight metabolites within nodes included two correlations and six negative correlations (Fig. 7E). In the correlation network of OP/SHAM, two proteins and two metabolites within nodes included two negative correlations (Fig. 7F).

**Table 4:** Correlation statistics of significant differential proteins and metabolites between GSK/OVX and OVX/SHAM

Group	Protein	Metabolites	Coefficient	***P***-value	Lable
GSK/OP	Mecp2	(3-Carboxypropyl)trimethylammonium cation	0.995	0.045	pos
	Slc1a3	L-Histidine	−0.997	0.045	neg
	Svs3b	Isobutyric acid	0.995	0.045	pos
	D3ZAT0	Riboflavin	−0.995	0.045	neg
	Rbm27	Purine	− 0.994	0.045	neg
	Jchain	1-Oleoyl-sn-glycero-3-phosphocholine	−0.998	0.045	neg
	Prps2	Oleic acid	−0.995	0.045	neg
	Aldh3b1	1-Stearoyl-sn-glycerol 3-phosphocholine	−0.995	0.045	neg
OP/SHAM	Tubb2a	L-Aspartate	−0.999	0.009	neg
	Kng2	DL-Methionine sulfoxide	−0.999	0.009	neg

### Integrated analysis of metabolic pathways involved in differential proteins and differential metabolites

**Fig. 8:**
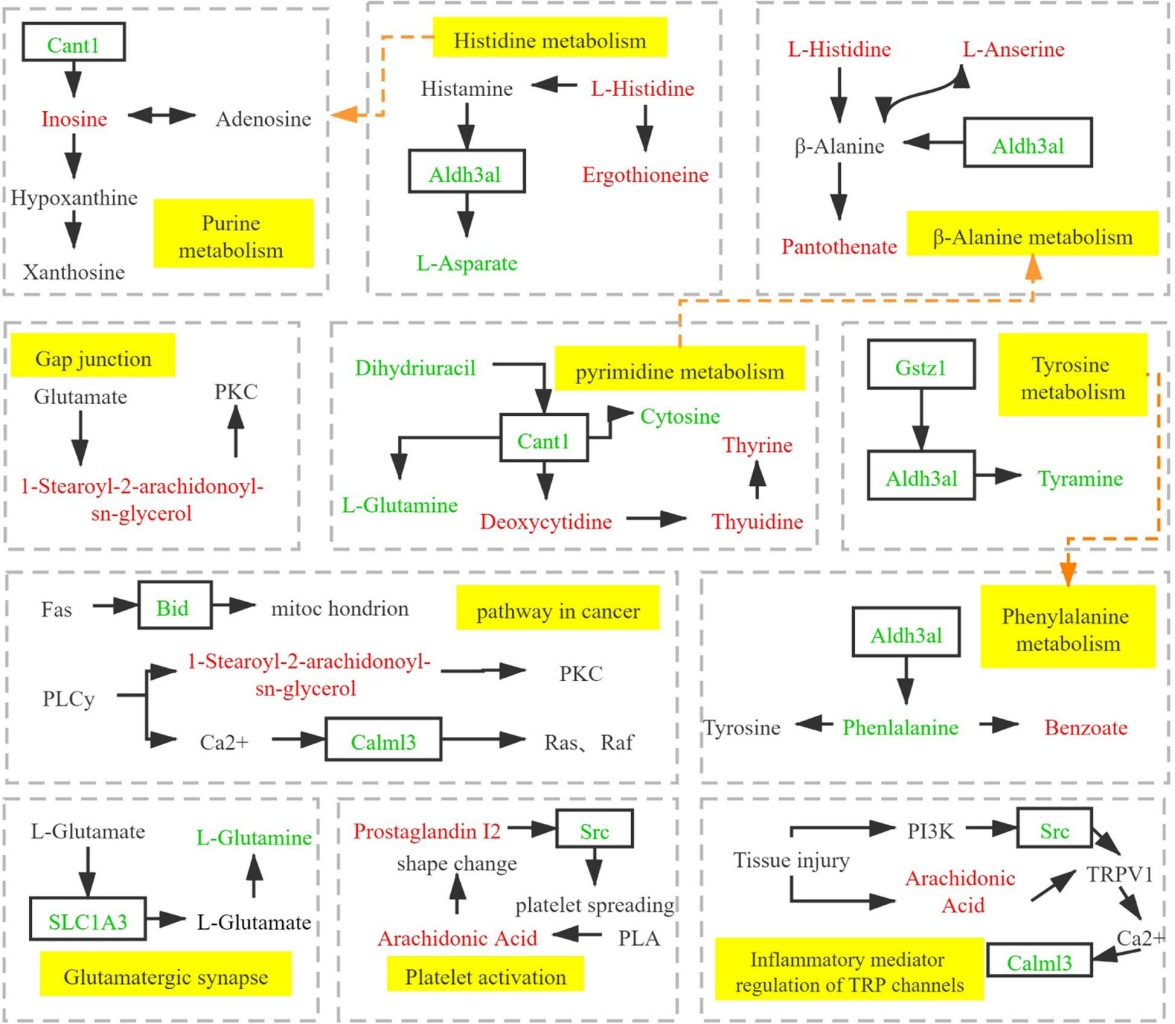
Figure showing common metabolic pathways of differential proteins and metabolites between GSK/OP and OP/SHAM. The boxes contain differentially expressed proteins. Red represents the upregulated expression of differential proteins or metabolites. Green shows the downregulated expression of proteins or metabolites. However, yellow depicts metabolic pathways involved in both differential metabolites and proteins

According to the statistics of the common metabolic pathways after GSK treatment and castration, differential proteins and metabolites jointly regulate 11 metabolic pathways. These include purine metabolism, pyrimidine metabolism, histidine metabolism, beta-alanine metabolism, inflammatory mediator regulation of TRP channels, platelet activation, tyrosine metabolism, cancer pathways, phenylalanine metabolism, glutamatergic synapses, and gap junctions, among others (Table 5). Among them, purine metabolism is correlated with histidine metabolism, beta-alanine metabolism is corelated with pyrimidine metabolism, and tyrosine metabolism is correlated with phenylalanine metabolism (Fig. 8).

**Table 5:** Differential proteins and metabolites enriched by the common metabolic pathways in GSK/OVX and OVX/SHAM. * represents a protein or metabolite with differential expression trends between GSK/OP and OP/SHAM

**No.**	**Metabolic pathway**	**Enriched protein**				**Enriched metabolites**			
		**GSK/OP**		**OP/SHAM**		**GSK/OP**		**OP/SHAM**	
1	Purine metabolism	*Cant1	↓	*Cant1	↑	L-Glutamine	↓	Xanthosine	↓
		Prps	↓	Enpp3	↑	Inosine	↑	Glycine	↑
						Adenosine	↓		
2	Tyrosine metabolism	*Gstz1	↓	*Gstz1	↑	*Tyramine	↓	Maleic acid	↑
		*Aldh3b1	↓	*Aldh3b1	↑			*Tyramine	↑
3	Pyrimidine metabolism	*Cant1	↓	*Cant1	↑	Dihydrouracil	↓	2'-Deoxyuridine	↓
						*Thymidine	↑	*Thymine	↓
						L-Glutamine	↓	*Thymidine	↓
						*Thymine	↑	*Deoxycytidine	↓
						*Deoxycytidine	↑	*Cytosine	↑
						*Cytosine	↓		
4	Histidine metabolism	*Aldh3b1	↓	*Aldh3b1	↑	*L-Aspartate	↓	L-Glutamate	↑
						*Ergothioneine	↑	*L-Aspartate	↑
						*L-Histidine	↑	*L-Histidine	↓
						*L-Anserine	↑	*L-Anserine	↓
						3-Methylhistidine	↑	*Ergothioneine	↓
5	beta-Alanine metabolism	*Aldh3b1	↓	*Aldh3b1	↑	Dihydrouracil	↓	*L-Aspartate	↑
						*L-Aspartate	↓	*Pantothenate	↑
						*L-Histidine	↑	*Histidine	↓
						*Pantothenate	↑	*L-Anserine	↓
						*L-Anserine	↑		
6	Pathways in cancer	Calml3 *Bid	↓ ↓	Fn1 Kng2 *Bid Cdk4	↑ ↑ ↑ ↑	1-Stearoyl-2-arachidonoyl-sn-glycerol	↑	L-Malic acid	↑
7	Platelet activation	Src	↓	Rlc-a	↑	*Arachidonic Acid (peroxide free)	↑	*Arachidonic Acid (peroxide free)	↓
				Fgb	↓				
				Col1a1	↓	1-Stearoyl-2-arachidonoyl-sn-glycero	↑		
				Col1a2	↓	Prostaglandin I2	↑		
8	Inflammatory mediator regulation of TRP channels	Calml3	↓	Kng2	↓	*Arachidonic Acid (peroxide free)	↑	*Arachidonic Acid (peroxide free)	↓
		Src	↓			1-Stearoyl-2-arachidonoyl-sn-glycerol	↑		
9	Phenylalanine metabolism	*Aldh3b1	↓	*Aldh3b1	↑	Hippuric acid	—	*Benzoic acid	↓
						*Benzoic acid	↑	*DL-Phenylalanine	↑
						*DL-Phenylalanine	↓		
10	Glutamatergic synapse	*Slc1a3	↓	*Slc1a3	↑	L-Glutamine	↓	L-Glutamate	↑
						1-Stearoyl-2-arachidonoyl-sn-glycerol	↑		
11	Gap junction	Src	↓	Tubb2a	↓	1-Stearoyl-2-arachidonoyl-sn-glycerol	↑	L-Glutamate	↑

### Protein levels of Slc1a3, Aldh3b1, Cant1, Gstz1, Bid decreased after treatment with GSK

**Fig. 9:**
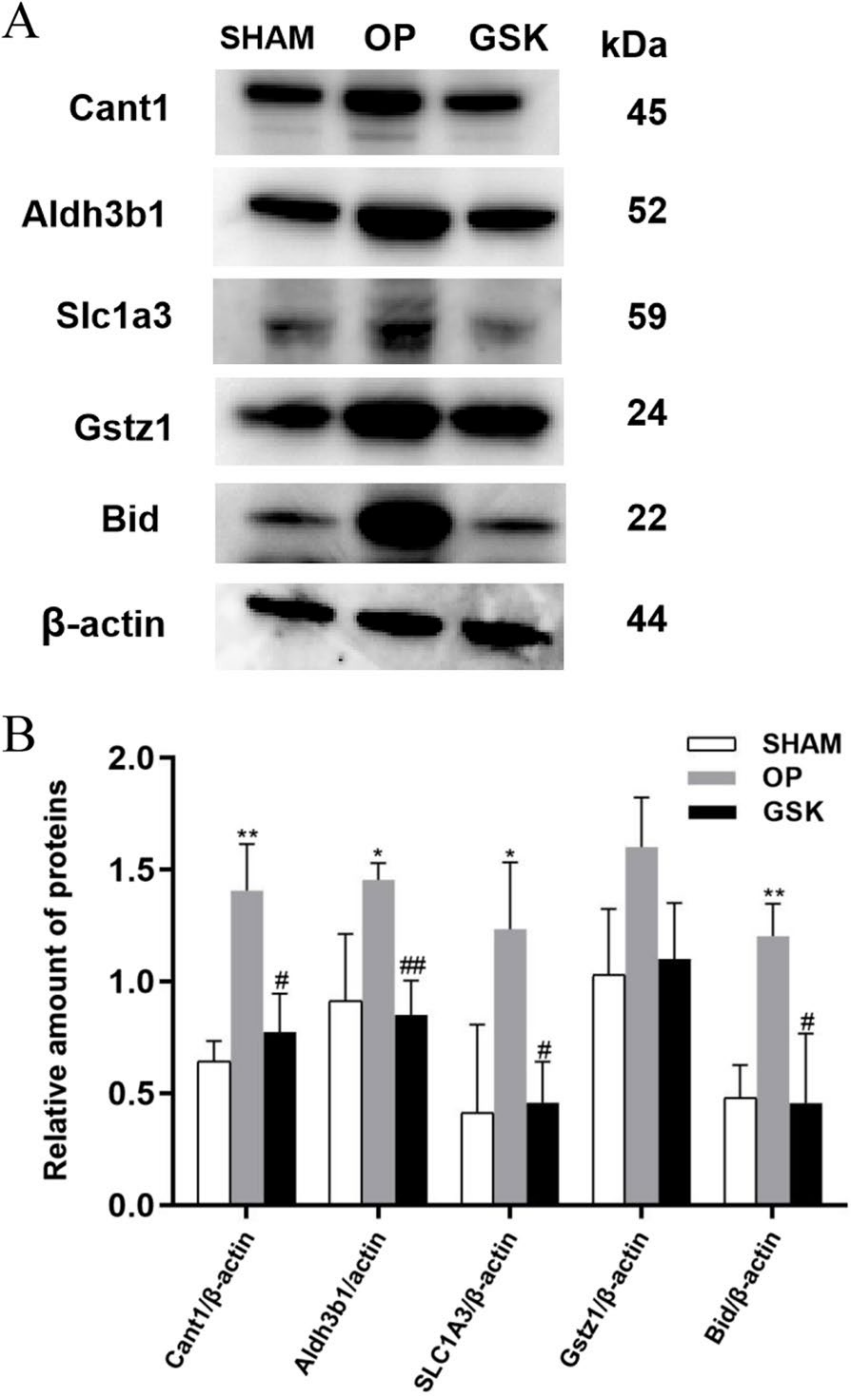
Expression of differential proteins in lumbar samples from the three groups. **A** Western blotting analyses of protein levels in lumbar in the SHAM, OP and GSK group. The image has been cropped and the original images can be found in supplementary material (Fig. 9C, Fig. 9D). The samples derived from the same experiment and blots were processed in parallel. **B** Protein levels in the lumbar of different rat groups. **p < 0.01 vs. the sham group; *p < 0.05 vs. the sham group; ^##^ *p* < 0.01 vs. the OP group; ^#^ *p* < 0.05 vs. the OP group

Based on the combined analysis of proteomics and metabonomics, we carried out western blot to verify the expression changes of five differential proteins. There were differences in levels of Slc1a3, Aldh3b1, Cant1, Gstz1, Bid among the groups (Fig. 9A,B). The expression levels of Slc1a3, Aldh3b1, Cant1, Bid in the OP group were increased significantly (*P* < 0.05 or *P* < 0.01) compared with those in the SHAM, and decreased significantly after 12 weeks of GSK treatment (GSK vs OP) (*P* < 0.05 or *P* < 0.01), which were consistent with the results of integrated analysis. In addition, GSK almost downregulated Gstz1, but the results showed no statistical significance. (*P* = 0.061).

## Discussion

With the increasing incidence of osteoporosis each year, the disease has become a worldwide public health and social problem [26]. Exploring the mechanism of GSK and other TCM can provide greater advantages and theoretical supplementation for TCM treatment and prevention of osteoporosis. Clearly, proteins and small molecule metabolites are closely associated with the occurrence of diseases. Thus, the combination of proteomics and metabonomics deserves our matched attention.

In this study, micro scanning CT data of bone trabecula showed that GSK capsules increased bone density and improved bone microarchitecture in OP rats. The specific molecular mechanism of GSK in the prevention and treatment of OP has not yet to be explained. Based on the combined analysis of proteomics and metabonomics, the results showed that the expression trends of differential proteins, i.e., Cant1, Gstz1, Aldh3b1, Bid, and Slc1a3, in the common metabolic pathway of differential proteins and metabolites between GSK/OP and OP/SHAM were different. Importantly, they were corrected in the GSK group, and we verified them through experiments. Cant1 is involved in purine and pyrimidine metabolism. Aldh3b1 is involved in the metabolism of tyrosine, histidine, beta-alanine, and phenylalanine. After GSK treatment, Slc1a3 was strongly correlated with L-histidine (*P* = 0.045), and Aldh3b1 was significantly correlated with 1-stearoyl-sn-glycerol 3-phosphocholine (*P* = 0.045). Combined with proteomic analysis, Gstz1 and Aldh3b1 were located in the functional nodes of the protein–protein interaction network. Additionally, the expression trend of 12 metabolites (tyramine, thymidine, deoxycytidine, cytosine, L-aspartate, ergothioneine, L-histidine, L-anserine, pantothenate, arachidonic acid (peroxide-free), benzoic acid, and DL-phenylalanine) differed in the common enrichment metabolic pathway between GSK/OP and OP/SHAM. The above metabolic pathways are primarily involved in nucleotide metabolism, amino acid metabolism, the immune system, cell processes, and other major signaling pathways, indicating that GSK may treat osteoporosis in rats through these.

Uric acid (UA) is the final product of purine metabolism in the human body. It is mainly decomposed from nucleic acids by enzymes or from other purine compounds metabolized by cells. UA is a reducing substance in the human body, participating in redox reactions and scavenging oxygen free radicals, thereby inhibiting oxidative stress [27, 28]. Several studies have reported that serum UA is involved in the pathogenesis of OP by affecting oxidative stress and inflammatory cascades [29]. Higher serum UA levels appear to be protective from bone loss in peri- and postmenopausal women [30]. Cant1, a calcium-activated nucleotide, is essential for glycosaminoglycan synthesis in cartilage [31]. In this study, Cant1, the key differential protein, was involved in purine metabolism, which downregulated after GSK treatment.

Amino acid metabolism is crucial for life activities. As the basic unit of macromolecular proteins, amino acids are essential for the normal metabolism of the human body [32]. Previous studies showed that amino acid metabolism was important to osteoporosis [33, 34]. In the correlation analysis, the significant differential proteins and metabolites indicate a strong correlation with histidine metabolism and β-alanine metabolism. During childhood, histidine is an essential amino acid in the human body. In adulthood, histidine can be synthesized by the human body, thereby making it nonessential. Histamine is formed under the action of histidine decarboxylase and is a factor that regulates inflammation and allergic reactions [35]. An increase in the amount of bone cortex and bone minerals was found in mice deficient in histamine synthesis, revealing that a lack of histamine may increase the bone density and bone formation of histamine-deficient mice [36].

The results showed that after treatment with GSK, Aldh3b1 in the histidine metabolism pathway was downregulated. L-aspartate was also downregulated. Ergothioneine, L-histidine, and L-anserine were upregulated. β-alanine plays a key role in bone metabolism, mainly by improving the production of insulin and insulin-like growth factor-1, as well as the synthesis of collagen and muscle proteins. Studies have shown that β-alanine may be a potential biomarker of osteoporosis, as β-alanine levels increase significantly in postmenopausal osteoporosis patients with low bone density [37]. Tyrosine is an important amino acid in the body and is the main raw material for the synthesis of thyroxine [38]. Previous studies have suggested that GSK may participate in bone metabolism by regulating tyrosine metabolism and differential proteins. It is reported that tyrosine metabolism is significantly related to osteoporosis [39]. In addition, Aldh3b1 is involved in the metabolism of tyrosine, histidine, beta-alanine, and phenylalanine. We speculate that Aldh3b1 may be closely related to the metabolism of amino acids. Aldh3b1 is a member of the ALDH family. Its function is related to the scavenging of reactive oxygen species [40]. In postmenopausal women, the level of estrogen and antioxidants decreased with age. Meanwhile, the ROS that accumulated in the body could not be cleared in time, leading to oxidative stress and the damage of osteoblasts and osteocytes [41, 42]. ALDH can reduce oxidative stress through a variety of pathways involved in aldehyde metabolism [43].

In this study, the expression pattern of arachidonic acid (AA) in the platelet activation pathway and inflammatory mediator regulation of TRP channels changed after GSK treatment. Platelets are known to play an important role in inflammation and thrombosis [44]. Higher platelet counts are significantly associated with osteopenia and osteoporosis [45]. AA and its products play important roles in regulating the inflammatory response [46], vascular elasticity, platelet activation, and bone remodeling. AA belongs to the polyunsaturated fatty acid (PUFA) family, one of the indispensable free fatty acids in the human body [47]. According to the position of the first double bond, PUFAs can be divided into n-3 PUFAs and n-6 PUFAs. n-6 PUFAs mainly include linoleic acid and AA. Recent research highlights the potential role of PUFA in the bone protection [48–50]. The combined use of n-3 PUFA and E2 exerted synergistic bone-protective efficacy through the upregulation of RUNX2, an essential transcription factor for bone formation, as well as the suppression of bone-resorbing cytokine IL-1β [51].

## Conclusions

This study reports the significant changes of proteins and metabolites in osteoporotic rats treated with GSK capsules. Integrated proteomic and metabonomic analyses showed that the GSK capsules may regulate differential proteins and differential metabolites to participate in nucleotide metabolism, amino acid metabolism, immune processes, and general cellular processes. They affect bone metabolism via the above mentioned pathways and play a role in bone protection. This study provides a reference for the molecular mechanism of the GSK capsules in the treatment of osteoporosis. Follow-up studies are also needed to further explore the differential proteins and metabolites in these metabolic pathways in order to find new drug targets and mechanisms of action.

## Supplementary Information

**Additional file 1.**

## Data Availability

The data analyzed during the study are available from the corresponding author on reasonable request.

## Abbreviations

OP: Osteoporosis
GSK: Gushukang
TCM: Traditional Chinese medicine
OVX: Ovariectomy
VIP: Variable Importance for the Projection
IDA: Information dependent acquisition
PLS-DA: Partial least squares discriminant analysis
OPLS-DA: Orthogonal partial least squares
BMD: Bone mineral density
PCA: Principal component analysis
UA: Uric acid
AA: Arachidonic acid
PUFAs: Polyunsaturated fatty acids
