# Supplementary material for: Integrated proteomics and metabolomics analysis of lumbar in a rat model of osteoporosis treated with Gushukang capsules
Source: BMC Complement Med Ther. 2022 Dec 15;22:333. doi: 10.1186/s12906-022-03807-7 (PMC9756464; doi:10.1186/s12906-022-03807-7)
Supplement: Supplementary file 1 — Additional file 1. [file 12906_2022_3807_MOESM1_ESM.doc]

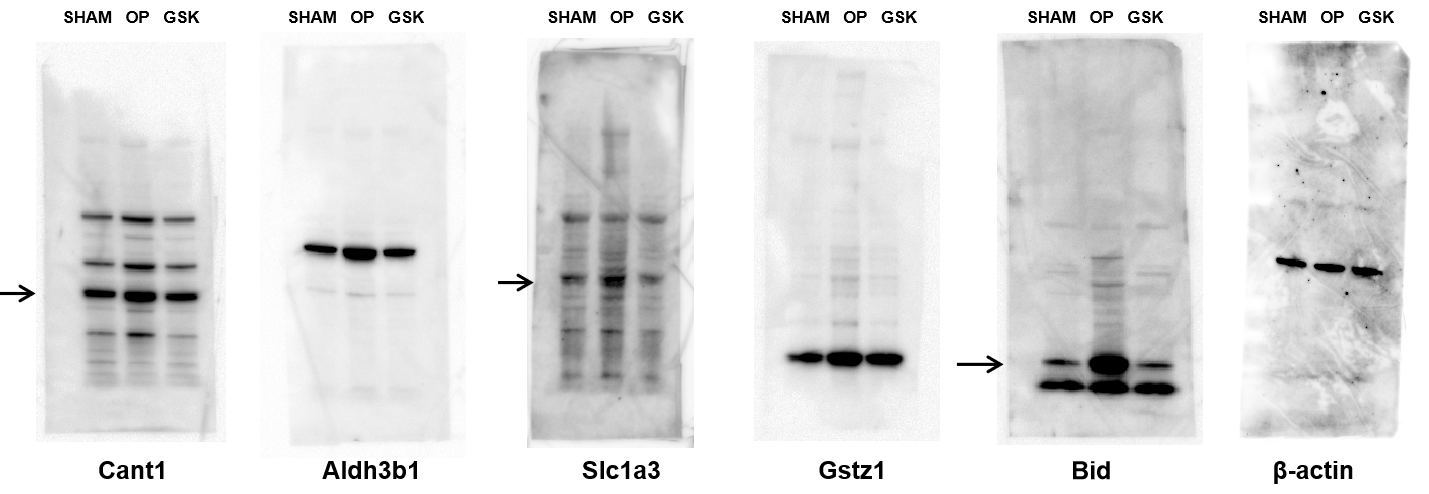


**Fig. 9C** Original image of differential expression proteins blot.


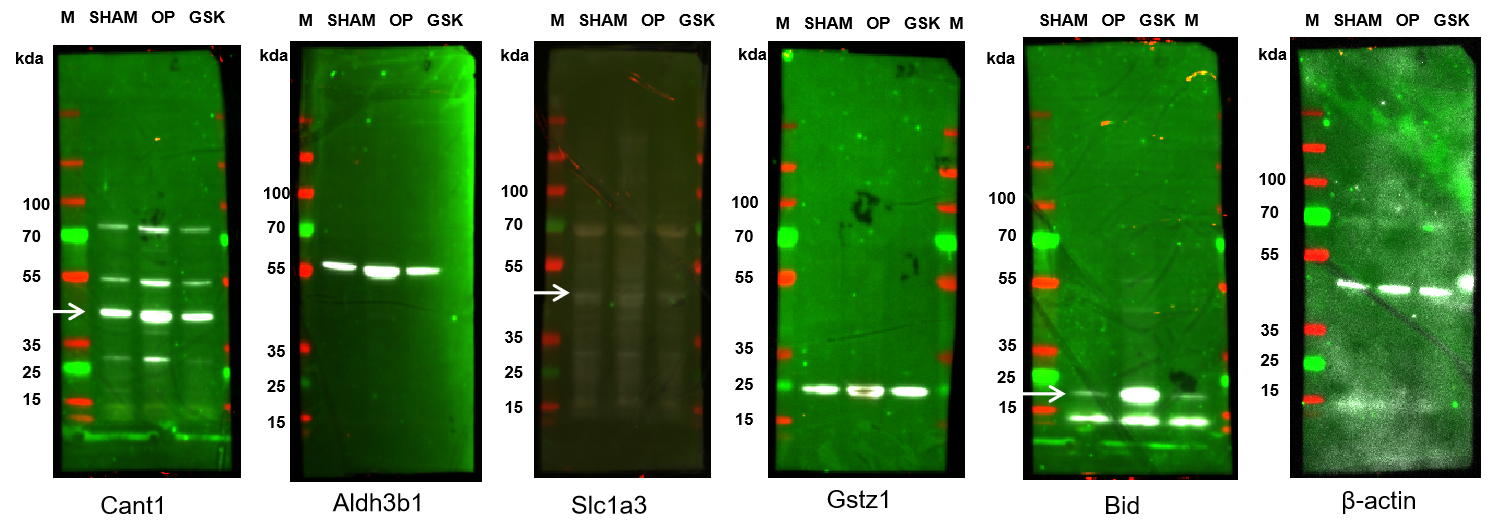


**Fig. 9D** Original image of differential expression proteins blot (In the mode of "chemi with markers") . This figure corresponds to Figure 9C. The markers are prestained and cannot be exposed using the chemiluminescence method, so the blots were exposed in the mode of "chemi with markers". Three images from three channels are stacked together to obtain the expected image with markers. In addition, the observed molecular weight of Slc1a3 was less than the expected molecular weight 59 kDa, the band of Slac1a3 which was used for statistical purposes in the experiments were the same size as that in the positive control (Rat brain)(Fig.9E).


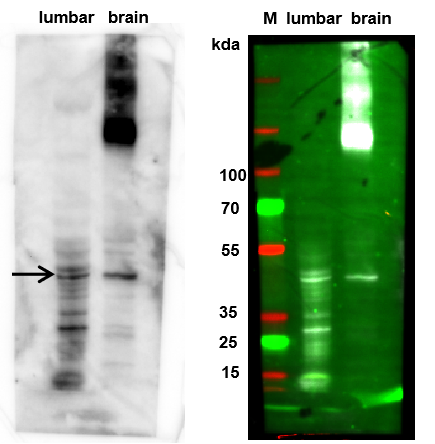


**Fig.9E** Original image of Slc1a3 protein blot in different samples. Brain is the positive control for Slc1a3.
